# Adopting and expanding ethical principles for generative artificial intelligence from military to healthcare

**DOI:** 10.1038/s41746-023-00965-x

**Published:** 2023-12-02

**Authors:** David Oniani, Jordan Hilsman, Yifan Peng, Ronald K. Poropatich, Jeremy C. Pamplin, Gary L. Legault, Yanshan Wang

**Affiliations:** 1 Department of Health Information Management, University of Pittsburgh, Pittsburgh, PA USA; 2 Department of Population Health Sciences, Weill Cornell Medicine, New York, NY USA; 3 Division of Pulmonary, Allergy, Critical Care & Sleep Medicine, University of Pittsburgh, Pittsburgh, PA USA; 4 Center for Military Medicine Research, University of Pittsburgh, Pittsburgh, PA USA; 5 Telemedicine & Advanced Technology Research Center, US Army, Fort Detrick, Frederick, MD USA; 6 Department of Surgery, Uniformed Services University, Bethesda, MD USA; 7 Virtual Medical Center, Brooke Army Medical Center, San Antonio, TX USA; 8 Intelligent Systems Program, University of Pittsburgh, Pittsburgh, PA USA; 9 Department of Biomedical Informatics, University of Pittsburgh, Pittsburgh, PA USA; 10 Clinical and Translational Science Institute, University of Pittsburgh, Pittsburgh, PA USA; 11 University of Pittsburgh Medical Center, Pittsburgh, PA USA

**Keywords:** Translational research, Health care

## Abstract

In 2020, the U.S. Department of Defense officially disclosed a set of ethical principles to guide the use of Artificial Intelligence (AI) technologies on future battlefields. Despite stark differences, there are core similarities between the military and medical service. Warriors on battlefields often face life-altering circumstances that require quick decision-making. Medical providers experience similar challenges in a rapidly changing healthcare environment, such as in the emergency department or during surgery treating a life-threatening condition. Generative AI, an emerging technology designed to efficiently generate valuable information, holds great promise. As computing power becomes more accessible and the abundance of health data, such as electronic health records, electrocardiograms, and medical images, increases, it is inevitable that healthcare will be revolutionized by this technology. Recently, generative AI has garnered a lot of attention in the medical research community, leading to debates about its application in the healthcare sector, mainly due to concerns about transparency and related issues. Meanwhile, questions around the potential exacerbation of health disparities due to modeling biases have raised notable ethical concerns regarding the use of this technology in healthcare. However, the ethical principles for generative AI in healthcare have been understudied. As a result, there are no clear solutions to address ethical concerns, and decision-makers often neglect to consider the significance of ethical principles before implementing generative AI in clinical practice. In an attempt to address these issues, we explore ethical principles from the military perspective and propose the “GREAT PLEA” ethical principles, namely Governability, Reliability, Equity, Accountability, Traceability, Privacy, Lawfulness, Empathy, and Autonomy for generative AI in healthcare. Furthermore, we introduce a framework for adopting and expanding these ethical principles in a practical way that has been useful in the military and can be applied to healthcare for generative AI, based on contrasting their ethical concerns and risks. Ultimately, we aim to proactively address the ethical dilemmas and challenges posed by the integration of generative AI into healthcare practice.

## Introduction

Artificial Intelligence (AI) plays an ever-increasing role in our daily lives and has influenced fields from online advertising to sales and from the military to healthcare. With the ongoing AI arms race in the Russia-Ukraine War, it is expected that AI-powered lethal weapon systems will become commonplace in warfare^1^. Although AI has shown promise in numerous successful applications, there remains a pressing need to address ethical concerns associated with these applications. There are dire consequences if an AI system selects an incorrect target potentially killing non-combatants or friendly forces. Seeing the rapid emergence of AI and its applications in the military, the United States Department of Defense (DOD) disclosed ethical principles for AI in 2020^2^. This document emphasized five core principles, aiming for responsible, equitable, traceable, reliable, and governable AI^2^. In addition, the North Atlantic Treaty Organization (NATO) also released principles for the use of AI in military, including lawfulness, responsibility and accountability, explainability and traceability, reliability, governability, and bias mitigation^3^. The success of these ethical principles has also been demonstrated through their ability to adopt and embed AI mindfully, taking into account AI’s potential dangers, which the Pentagon is determined to avoid^4^. Clearly, prominent military organizations demonstrate a cautious approach toward adopting AI and are actively implementing measures to mitigate the risks associated with its potential malicious uses and applications.

On the other hand, AI has had a direct impact on the healthcare industry, with discussions ranging from the uses of AI as an assistant to medical personnel^5–7^ to AI replacing entire clinical departments^8,9^. The use and impact of AI in clinical Natural Language Processing (NLP) in the context of Electronic Health Records (EHRs) have been profound^10–13^. Similar to military organizations, the World Health Organization (WHO) has also released a document discussing the ethics and governance of AI for health^14^.

Generative AI, as the name suggests, refers to AI techniques that can be used to create or produce various types of new contents, including text, images, audio, and videos. The rate of development of generative AI has been staggering, with many industries and researchers finding its use in fields such as finance^15^, collaborative writing^16^, email communication^17^, and cyber threat intelligence^18^. Generative AI has also become an active area of research in the healthcare domain^19,20^, with applications such as clinical documentation^21^ and evidence-based medicine summarization^22^.

Despite many successful and promising AI applications, ethics has been one of the more controversial subjects of discussion in the AI community, with diverging views and a plethora of opinions^23,24^. Ethics deals with how one decides what is morally right or wrong and is one of the pivotal aspects that we, as the AI research community, have to consider carefully. Given the recent emergence of generative AI models and their initial enthusiasm in healthcare, our community must seriously consider ethical principles before integrating these techniques into practical use. The military and healthcare are notably similar in many ways, such as organizational structure, high levels of stress and risk, decision-making processes, reliance on protocols, and dominion over life and death. Given these parallels, successful implementation of ethical principles in military applications, and the lack of specific solutions to generative AI ethics in healthcare, we propose to adopt and expand ethical principles, from military to healthcare, to govern the application of generative AI in healthcare applications.

## What is generative artificial intelligence?

Generative AI refers to AI that is used primarily for generating data, often in the form of audio, text, and images. However, in this manuscript, we choose not to follow such a general definition and instead, focus on a particular type of generative AI. In this section, we describe “modern” generative AI, discuss why it is important, and compare it to the term that has become so popular—“AI.”

**Fig. 1:**
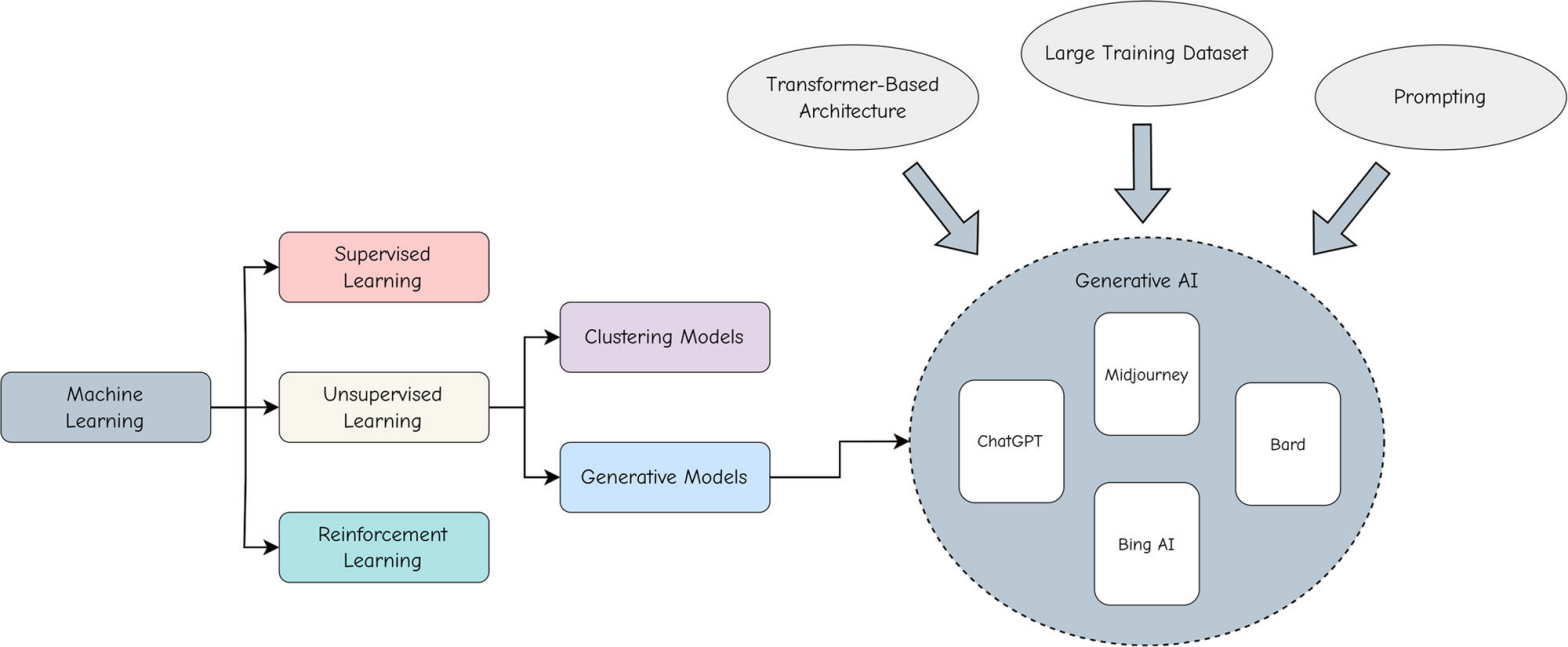
The relationship between general ML and modern generative AI. The figure provides an overview of ML subfields, establishes relationships among these subfields, and shows the path to generative AI.

Modern AI is dominated by Machine Learning (ML) methods, which leverage statistical algorithms and large amounts of data to gradually improve model performance. ML methods could roughly be classified into supervised, unsupervised, and reinforcement learning (Fig. 1). Supervised ML relies on labeled input (supervision), while unsupervised learning needs no human supervision. Reinforcement learning takes a different approach and, instead, attempts to design intelligent agents by rewarding desired behaviors and punishing undesired ones. Popular generative AI models are typically pre-trained in an unsupervised manner.

The pre-trained generative AI models could generate novel and diverse outputs, including, but not limited to, text, images, audio, or videos. Recently, the most popular generative AI model for language generation is ChatGPT^25^, which was reported to have an estimated 100 million monthly active users in January 2023^26^. The model architectures for ChatGPT, previously known as GPT-3.5^27^, and more recent GPT-4^28^, are built upon the design principles of its GPT^29^ (Generative Pre-trained Transformer) predecessors, GPT-2^30^ and GPT-3^31^. Many state-of-the-art generative AI models, also known as Large Language Models (LLMs), share a similar transformer-based architecture^32^.

The well-known generative AI models used for image generation from text prompts, such as Stable Diffusion^33^ and DALL-E 2^34^, employ a combination of the diffusion process^35^ and a transformer-based architecture similar to the one used in GPT models. All of the models are characterized by unsupervised training on very large datasets^36^. The same is true of models that generate videos.

Most of these generative AI models also rely on a method called prompting^37^, which lets users input a natural language description of a task and uses it as a context to generate useful information. This process is also sometimes referred to as in-context learning.

When referring to “modern” generative AI or simply generative AI, we are describing a transformer-based machine learning model trained in an unsupervised manner on extensive datasets and specifically optimized for generating valuable data through prompts. This description also aligns harmoniously with existing research and studies^38–40^.

While generative AI shows promising results, dangerous outcomes in healthcare can arise from a number of issues, including:

- Algorithmic bias^41,42^
- Hallucination^43,44^
- Poor commonsense reasoning^44,45^
- Lack of generally agreed model evaluation metrics^46,47^

All of these issues are common for generative AI in general, but more so in the healthcare domain, where algorithmic bias may result in the mistreatment of patients^48^, hallucination may carry misinformation^49^, poor commonsense reasoning can result in confusing interactions^50^, and lack of general and domain-specific metrics can make it difficult to validate the robustness of the AI system^51^. Furthermore, in the context of healthcare, there are concerns about leaking Protected Health Information (PHI)^52^ as well as lacking empathy to patients^53^.

Such concerns can also be present in other forms of AI, but given the practical differences present in generative AI, the risks become elevated. First, due to the interactive nature of generative AI, often paired with the ability to hold human-like dialogs (e.g., ChatGPT), it can make misinformation sound convincing. Second, since generative AI models combine various sources of large-scale data^36^, the risk of training on biased data sources increases. Third, the standard evaluation metrics, such as precision, recall, and F1 score, become difficult to use and are less likely to reflect human judgment^47^. Finally, due to its ease of use, generative AI can be widely adopted in many fields and domains of healthcare^49^, which naturally increases the aforementioned risks.

Overall, the importance of ethical considerations for generative AI in healthcare cannot be understated. From the human-centered perspective, the ultimate goal of generative AI is to enhance and augment human’s creativity, productivity, and problem-solving capabilities, which is well aligned with the goal of healthcare in improving patient care. If the generative AI system is not used ethically and does not reflect our values, its role as a tool for improving the lives of people will greatly diminish.

## AI applications in military vs. healthcare

With the increasing prevalence of AI, it has been in the best interest of military organizations to understand and integrate AI into their operations and strategies to be at the cutting edge of security and technology in conflict or emergency. Various military AI technologies for generative purposes have also been developed, including Intelligent Decision Support Systems (IDSSs) and Aided Target Recognition (AiTC), which assist in decision-making, target recognition, and casualty care in the field^54–56^. Each of these uses of AI in military operations reduces the mental load of operators in the field and helps them take action more quickly. Just as military uses of AI can save lives on the battlefield, AI can help save lives by assisting clinicians in diagnosing diseases and reducing risks to patient safety^57–59^. Uses of generative AI in healthcare help improve the efficiency of professionals caring for patients. Applications of generative AI in healthcare include medical chatbots, disease prediction, CT image reconstruction, and clinical decision support tools^60–63^. The benefits of such uses are two-fold, in that they can help healthcare professionals deliver a higher level of care to their patients, as well as improve the workload within clinics and hospitals.

People may question that developing AI models for military and healthcare purposes hinges on distinct ideological underpinnings reflecting unique priorities. In the military context, AI models are primarily designed to enhance the efficiency, precision, and strategic capabilities of both defensive and offensive operations. The focus is on applications such as surveillance, target recognition, cyber defense, autonomous weaponry, and battlefield analytics. Potential future uses of AI for offensive actions such as coordinating drone attacks may oppose any healthcare principle, yet is vital for the military strategy. The fundamental ideological perspective here is the protection of national security interests, force multiplication, and minimizing human risk in conflict zones.

On the other hand, the use of AI in healthcare is driven by the principles of enhancing patient care, improving health outcomes, and optimizing the efficiency of healthcare systems. The development of AI models in this sector aims to personalize treatments, improve diagnostic accuracy, predict disease progression, and streamline administrative tasks, among other uses. The central ideology is the betterment of human health and well-being. While we acknowledge the different ideological foundations in military and healthcare due to the contrasting objectives, we argue that both military and healthcare sectors illustrate a compelling convergence of priorities for the applications of AI.

Specifically, their shared focus on application validity, attention to practical implementation, and prioritization of a human-centered approach have emerged as significant commonalities. First, concerning application validity, both fields recognize the crucial importance of robust, reliable AI systems. These systems need to function accurately and rapidly under diverse, often challenging, conditions to fulfill their designated tasks, whether it identifies potential security threats in a complex battlefield or detects subtle abnormalities in medical images. Second, there is an evident emphasis on implementation. Beyond the theoretical development of AI models, the critical question for both sectors centers around how these models can be effectively incorporated into real-world systems, often involving multiple human and technological stakeholders. Finally, a human-centered perspective is paramount. This means ensuring that AI technologies augment, rather than replace, human decision-making capacities and are employed in ways minimizing potential harm. In healthcare, this involves developing AI applications that can improve patient outcomes and experience while supporting healthcare providers in their work. Thus, these three factors represent key shared priorities in the utilization of AI across military and healthcare contexts.

AI has been seamlessly woven into the military’s technology fabric for several decades, serving as the backbone for various advancements ranging from autonomous drone weapons to intelligent cruise missiles^64,65^. The track record of robust results and reliable outcomes in complex and high-risk environments implicitly engage with foundational ethical principles. The ethical guidelines established from military AI implementations have provided a road map for the incorporation of AI in healthcare scenarios. However, the integration of AI is relatively new to the healthcare sector, let alone generative AI, and ethical principles are neither widely implemented nor specifically designed for generative AI. While healthcare has begun to adopt generative AI technologies more recently^66^, there are immense opportunities for this field to glean ethical insights from the history of military application.

## Identifying ethical concerns and risks

A RAND Corporation study raised various concerns about the use of AI in warfare, shown in Figure 2.3 of the research report^67^. These concerns fall into the following categories: increasing risk of war, increased errors, and misplaced faith in AI. Although AI can allow personnel to make decisions and strategies more quickly, some experts consider this a downside, as actions taken without proper consideration could have serious repercussions, like increasing the risk of war^67^. International standards for warfare like the Law of Armed Conflict (LOAC) and Geneva Conventions lay out guidelines for target identification specifying that attacks must first distinguish between combatant and noncombatant targets before taking action to minimize harm to civilians^68,69^. Because combatants are not always identifiable visually, some claim that reading body language to differentiate a civilian from a combatant necessitates a Human-In-The-Loop (HITL) decision-making process^70^.

Maintaining data privacy for users of generative AI technologies is critical, as both patient data and military data are highly sensitive, and would be damaging if leaked^71^. If an AI implementation collects PHI, it should be secure against breaches, and any disclosures of this protected data must comply with Health Insurance Portability and Accountability Act (HIPAA) guidelines^72^. These implementations must experience few errors as healthcare is a safety-critical domain where the patient harm is unacceptable^73^, and errors in these systems or algorithms could cause more harm than any physician would be capable of, as many hospitals and clinics would be using the same systems and experiencing the same errors^74^.

**Fig. 2:**
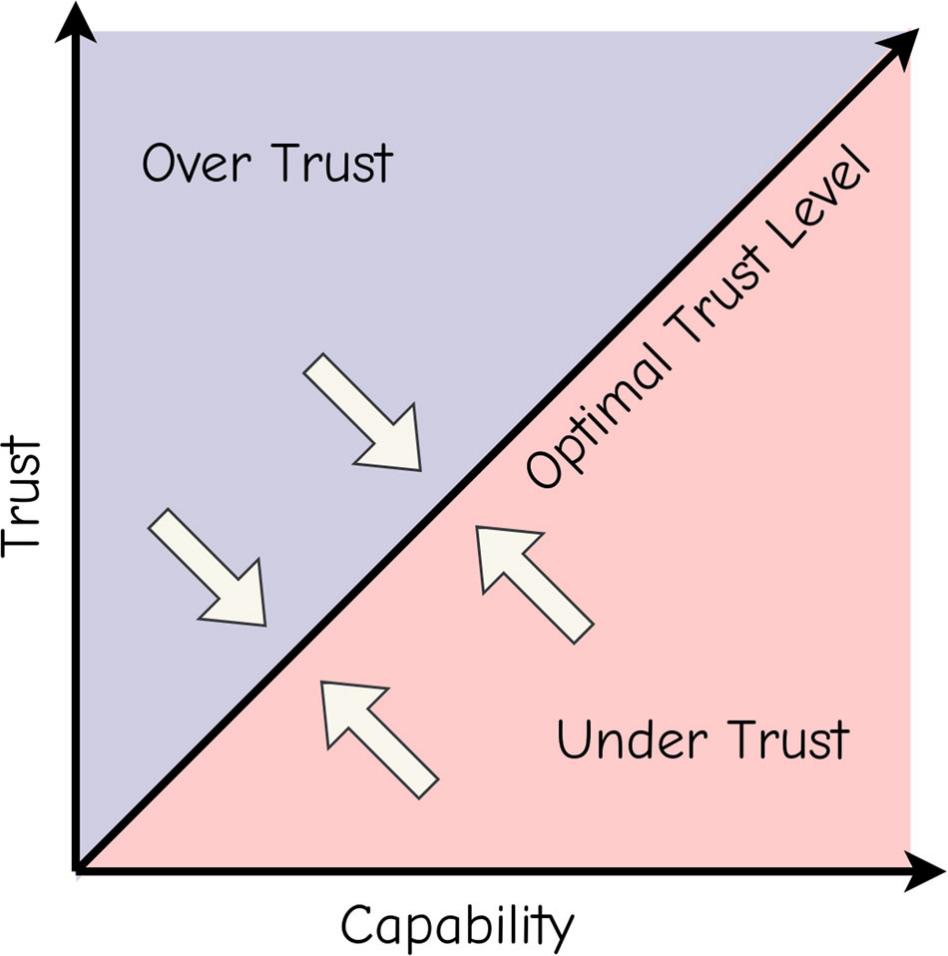
Optimization of trust in AI. The figure depicts the relationship between trust and capability. Too much trust in AI systems can lead to overuse of the AI when it is not in the best interest of patients or operators, and too little trust can lead to underuse of the system when it would be better to use it.

Additional concerns present in the military and healthcare are trust between humans and AI and the lack of accountability. When there exists human-and-AI collaboration to perform a task, trust must be optimal, as shown in Fig. 2. Too much trust in AI systems can lead to overuse of the AI when it is not in the best interest of patients or operators^75^, and too little trust can lead to underuse of the system when it would be better to use it^76^. In both situations, the root cause is operators not knowing the capabilities and limitations of the systems they interact with^77^. Misuse can lead to non-typical errors, such as fratricide in the military or patient harm in a hospital^78,79^. While the AI must be transparent in its decision-making, the use of AI must be accompanied by sufficient education on the use and limitations of AI systems so that operators are less likely to make dangerous errors. A lack of accountability can possibly arise in military or healthcare use of generative AI because military operators or clinicians do not have direct control over the actions determined by the AI.

In the same research report by RAND Corporation^67^, authors showed (Figures 7.8, 7.9 in the report) that the general public views autonomous systems taking military action with human authorization favorably while strongly disagreeing with combat action without human authorization. The parallel can be drawn with healthcare, where patients express concerns over the use of AI for medical purposes without human (e.g., physician, nurse, etc.) involvement^80^. These results could be due to the perceived lack of accountability, which is considered something that could entirely negate the value of AI, as a fully autonomous system that makes its own decisions distances military operators or clinicians from the responsibility of the system’s actions^81^. In healthcare, it is critical that the systems are transparent due to their proximity to human lives and that patients understand how clinicians use these recommendations. The burden of accountability in the healthcare sector falls to both the clinicians and the developers of the AI systems, as the decisions made are a product of the algorithm, and the use of these recommendations falls to the clinicians^82^.

Finally, ethical concerns of equity, autonomy, and privacy regarding the use of generative AI must also be considered. In healthcare settings, biased algorithms or biased practices can lead to certain patient groups receiving lower levels of care^83^. Biased outcomes could be due to biased algorithms, poor data collection, or a lack of diversity^84^. There must be minimal bias in developing AI systems in healthcare, both in the algorithm and the data used for training. Furthermore, if known, the sources of bias must also be disclosed to ensure transparency and prevent inappropriate use. The issue of human autonomy when developing generative AI is especially pertinent in healthcare, as both patient and clinician autonomy must be respected^85^. It is crucial that a framework is accepted to prevent any data breaches and ensure security measures are up to date and robust.

These risks and ethical concerns surrounding generative AI in military and healthcare applications necessitate principles for the ethical use of AI. One of the earliest sets of principles published for responsible development and use of AI comes from Google, who did so in response to their employees petitioning their CEO as they disagreed with Google working with the DOD on Project Maven to assist in identifying objects in drone images^86,87^ in 2018. These principles outline how Google will develop AI responsibly and state what technologies they will not create, like those that cause harm or injure people, provide surveillance that violates international policies, and any technologies that go against international law and human rights^88^. By examining the differences and similarities between risks and ethical concerns in military and healthcare applications of generative AI, we can establish guiding principles for the responsible development and use of generative AI in healthcare.

## GREAT PLEA ethical principles for generative AI in healthcare

As AI usage has spread throughout the military and other fields, many organizations have recognized the necessity of articulating their ethical principles and outlining the responsibilities associated with applying AI to their operations. There are several ethical principles for AI published by various organizations, including the U.S. Department of Defense^2^, NATO^3^, the American Medical Association (AMA)^89^, the World Health Organization (WHO)^14^, and the Coalition for Health AI (CHAI)^90^. The AI ethical principles for DOD and NATO are similar, with NATO having an added focus on adherence to international law. For the development of AI for healthcare, the WHO has published its own ethical principles, including protecting human autonomy, human well-being and safety, transparency and explainability, responsibility and accountability, inclusiveness, and responsive development. Similarly, the AMA promotes AI systems that should be user-centered, transparent, reproducible, avoid exacerbating healthcare disparities, and safeguard the privacy interests of patients and other individuals. Finally, there is the Blueprint for an AI Bill of Rights published by the U.S. Office of Science and Technology Policy (OSTP)^91^, which has provisions for AI systems to be safe and effective, protected against algorithmic discrimination, protect user data, have accessible documentation, and offer human alternatives.

Among the various sets of principles, we see common themes such as accountability and human presence. The DOD and NATO both emphasize the importance of integrating human responsibility into the development and life cycle of an AI system, as well as ensuring these systems are governable to address errors that may arise during use. The AMA and WHO policies both highlight a human-centered design philosophy protecting human autonomy and explicitly mention the need for inclusiveness and equity in the healthcare use of AI to prevent care disparity. These principles each provide unique perspectives for developing AI for healthcare use. However, no set of principles encompasses all ethical concerns that healthcare providers or patients may have^92^. Adopting the principles of the DOD and NATO is advantageous due to each principle’s practical definition. These principles are outlined with a focus on what actions can be taken by personnel developing AI systems, and how end-users would interact with the systems.

The existing principles establish a good foundation for the ethical development and utilization of AI in healthcare. However, action must be taken to tailor these principles for generative AI. By examining the risks and concerns surrounding the use of generative AI in healthcare, comparing them to the risks and concerns of generative AI in the military, and by expanding these principles, we can have a set of principles that fulfill our needs^93^. Therefore, we use DOD and NATO guidelines as the starting point for the set of ethical principles, and expand them to meet the needs in healthcare. The expansion is done by incorporating principles that support the betterment of mankind rather than defeating adversaries.

**Fig. 3:**
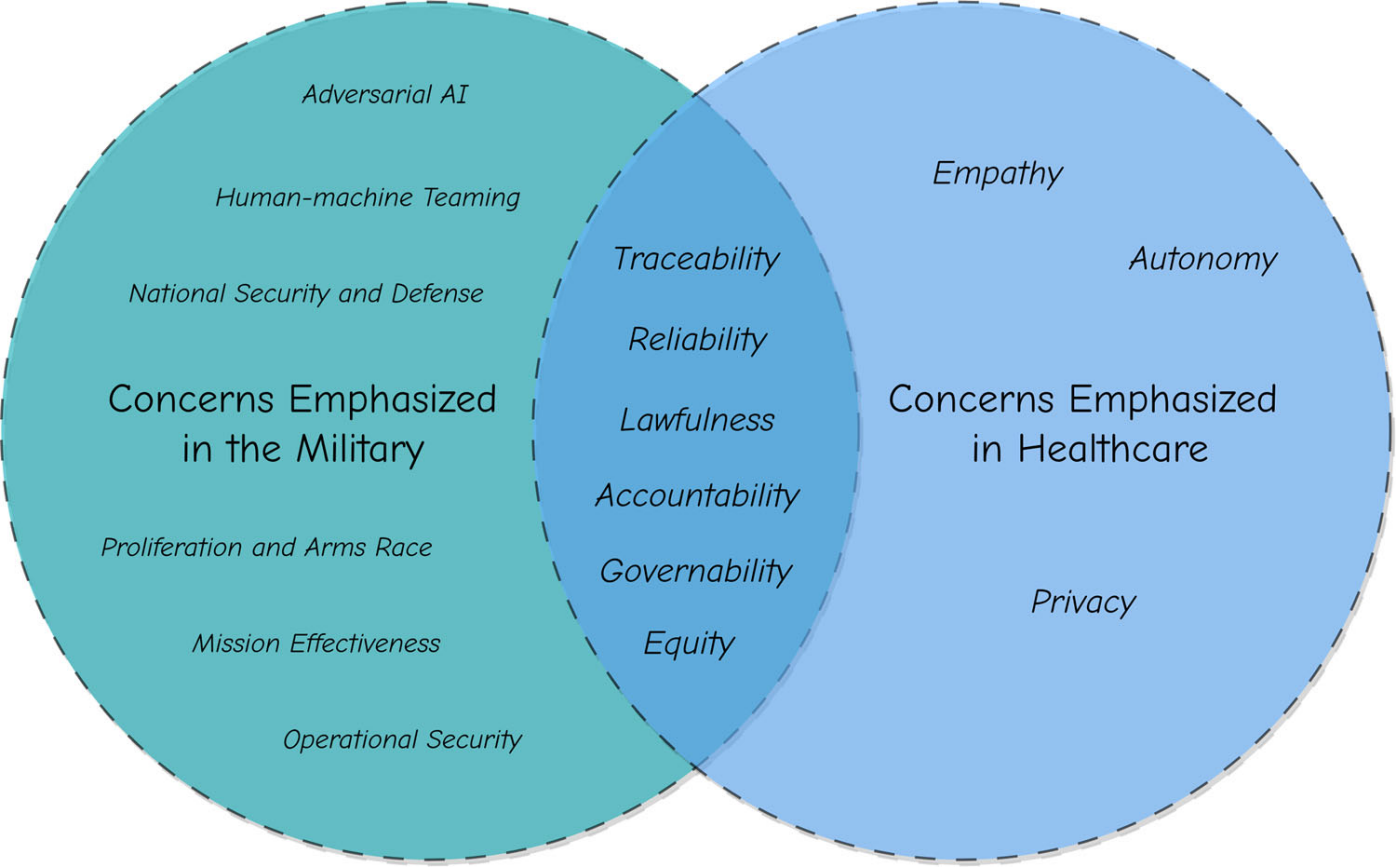
Adoption and expansion of existing ethical principles from military to healthcare. The figure illustrates the commonalities and differences in ethical principles between military and healthcare. In our assessment, traceability, reliability, lawfulness, accountability, governability, and equity are the ethical principles that both fields have in common. At the same time, ethical principles, such as empathy and privacy, are emphasized in healthcare, whereas ethical principles, such as national security and defense, are emphasized in the military.

Figure 3 shows the framework that we used for adopting and expanding ethical principles, established by various organizations, for the healthcare applications of AI. Where similarities are present in the concerns between military and healthcare use of generative AI, it is possible to adopt principles for use, such as traceability, reliability, lawfulness, accountability, governability, and equity. In instances when healthcare has unique circumstances or requires additional nuance, the principles related to those matters must be expanded to fit into the world of medicine, such as empathy, autonomy, and privacy. There are many concerns specific to the military that are unsuitable for forming ethical principles in healthcare, such as national security and defense, mission effectiveness, operational security, adversarial AI^94^, human-machine teaming, rules of engagement, rapid deployment and adaptation, and proliferation and arms race. Figure 3 also shows some of these concerns (a non-exhaustive list), which we included to highlight that the adoption or expansion of principles must be based on shared concerns. Furthermore, we want to emphasize the need for having safeguards and methods to detect and mitigate military-specific properties of AI deployed in healthcare settings, by including governability, accountability, and traceability.

A detailed mapping of the proposed ethical principles to those used by DOD, NATO, and WHO guidelines is shown in Table 1. As shown in the table, all principles, except for privacy, empathy, and autonomy, directly align with either DOD or NATO guidelines. In cases where the principle indirectly aligns with our proposed principles, Table 1 uses a star (^*^) prefix. As for privacy, empathy, and autonomy, despite not being related to the ethical principles in military organizations (i.e., DOD and NATO), WHO guidelines directly or indirectly align with all three. Their inclusion was also due to the quality of betterment of mankind and mitigation of concerns specific to healthcare.

**Table 1:** Alignment of GREAT PLEA ethical principles with DOD, NATO, and WHO guidelines.

Principle	DOD	NATO	WHO
Governability	Governable	Governability	*Promote AI that is responsive and sustainable
Reliability	Reliable	Reliability	*Promote human well-being, human safety, and the public interest
Equity	Equitable	Bias mitigation	Ensure inclusiveness and equity
Accountability	^*^Responsible	Responsibility and accountability	Foster responsibility and accountability
Traceability	Traceable	Explainability and traceability	Ensure transparency, explainability, and intelligibility
Privacy	None	None	*Ensure transparency, explainability, and intelligibility
Lawfulness	None	Lawfulness	*Promote AI that is responsive and sustainable
Empathy	None	None	*Promote human well-being, human safety, and the public interest
Autonomy	None	None	Protect autonomy

^*^mark: indirectly aligned principle

**Fig. 4:**
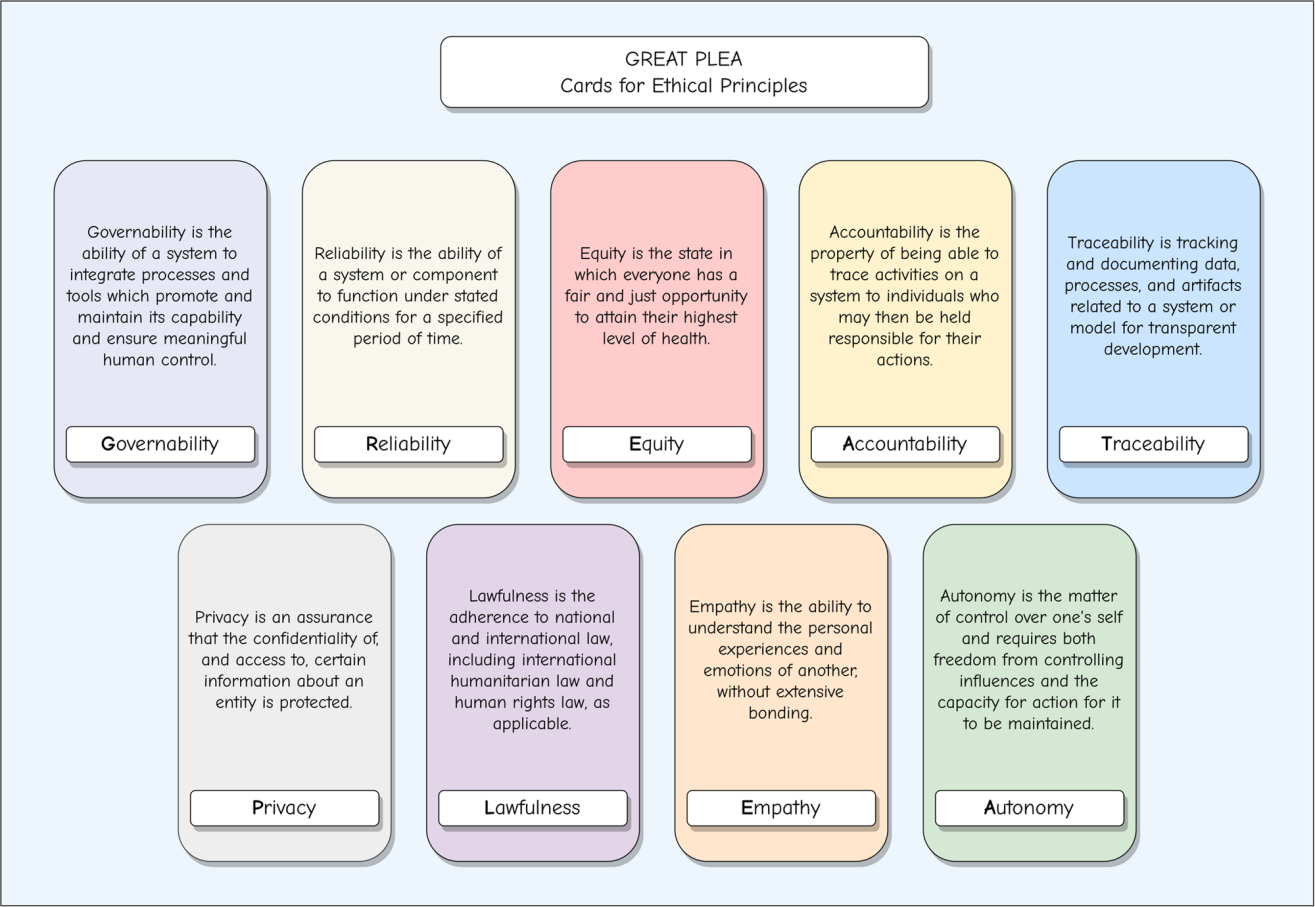
GREAT PLEA cards for ethical principles. We propose the “GREAT PLEA” ethical principles for generative AI in healthcare, namely Governability, Reliability, Equity, Accountability, Traceability, Privacy, Lawfulness, Empathy, and Autonomy. The GREAT PLEA ethical principles demonstrate our great plea for the community to prioritize these ethical principles when implementing and utilizing generative AI in practical healthcare settings.

In summary, we propose the “GREAT PLEA” ethical principles for generative AI in healthcare, namely Governability, Reliability, Equity, Accountability, Traceability, Privacy, Lawfulness, Empathy, and Autonomy. The GREAT PLEA ethical principles demonstrate our great plea for the community to prioritize these ethical principles when implementing and utilizing generative AI in practical healthcare settings. Fig. 4 shows the summary cards for the GREAT PLEA ethical principles. In the following, we will delve into a comprehensive explanation of each individual principle.

### Governability

Governability is the ability of a system to integrate processes and tools which promote and maintain its capability and ensure meaningful human control^95^. Standards for the governability of AI systems, as established by the DOD and NATO, emphasize the importance of ensuring that while AI systems fulfill their intended functions, humans must retain the ability to identify and prevent unintended consequences. In the event of any unintended behavior, human intervention to disengage or deactivate the deployed AI system should be possible. These standards can be adopted for the use of generative AI in healthcare. Due to the potential of widespread implementation of generative AI systems, where numerous hospitals may be using the same systems, these standards must be considered^74^. Suppose a generative AI system, deployed across multiple clinics, poses a risk of harm to a patient. In that case, it is crucial to recognize that numerous patients across clinics could be vulnerable to the same error. Risk to patients amplifies as healthcare expands to patient homes with remote patient monitoring or with online tools outside the clinic. Ideally, humans, whether they develop or implement the system, should possess the capability to deactivate it without disrupting the regular patient care activities in the clinics. There must be explicit guidelines for monitoring generative AI systems for potential errors, deactivation to prevent more damage when an error occurs, remedying errors, and interaction to reduce operator errors. With these guidelines in place, personnel in charge of the system can quickly be notified of any unintended behavior and respond quickly and appropriately.

### Reliability

Reliability is the ability of a system or component to function under stated conditions for a specified period of time^96^. The proximity of generative AI to patient well-being necessitates standards for reliability to minimize potential errors that could lead to accidents^43^. The generative AI models should have explicit and well-defined clinical use cases. A generative AI model designed for disease prediction needs to have a clear definition of the use situation and patient criteria. In addition, such generative AI models should be safe, secure, and effective throughout their life cycles. Generative AI models should be demonstrated to be at least as safe as human decision making alone and not cause undue harm. Existing generative AI models suffer from hallucination and output variations, undermining their ability to produce reliable outputs. These shortcomings can adversely affect the trust between physicians and generative AI systems. Adopting the DOD’s principle for reliability can establish use cases for AI applications and monitor them during development and deployment to fix system failures and deterioration. Having a thorough evaluation and testing protocol against specific use cases will ensure the development of resilient and robust AI systems, and help minimize system failures as well as the time needed to respond to these errors.

### Equity

Equity is the state in which everyone has a fair and just opportunity to attain their highest level of health^97^. Due to the importance of health equity and the ramifications of algorithmic bias in healthcare, we call for adjustments to this principle. There already exists inequity in healthcare. The generative AI models, that naturally have elevated data bias risks due to their pre-training on massive datasets, should not exacerbate this inequity for marginalized, under-represented, socioeconomically disadvantaged, low education, or low health literacy groups^98^, but rather incorporate their unique social situations into future AI models to insure equity. Generative AI must be developed with efforts to mitigate bias by accounting for existing health disparities. Without this consideration, generative AI systems could erroneously recommend treatments for different patients^99^. Expansion of the principle for equity must set standards for evaluation metrics of algorithmic fairness so that deployed AI systems will not reinforce healthcare disparity.

### Accountability

Accountability is the property of being able to trace activities on a system to individuals who may then be held responsible for their actions^100^. To ensure accountability and human involvement with AI in healthcare, the principle of Responsibility and Accountability outlined by NATO^3^ states that they will develop AI applications mindfully and integrate human responsibility to establish human accountability for actions taken by or with the application. A study of patient attitudes toward AI showed the importance of accountability in gaining patient trust when using AI in healthcare^101^. This assurance of accountability is crucial when a clinician is using generative AI to help treat a patient, as without proper measures for human accountability, the patient may feel that the clinician is not invested in the care they are delivering^102^. We can adopt this principle for the ethical use of generative AI in healthcare, and ensure that human involvement is maintained when more powerful generative AI systems, such as ChatGPT or generative AI-based clinical decision support systems, are used in patient care.

### Traceability

Traceability is tracking and documenting data, processes, and artifacts related to a system or model for transparent development^103^. Addressing the issue of optimizing trust between healthcare professionals and the AI they interact with can be done by adopting the principle of traceability. This way, the personnel working with AI will understand its capabilities, developmental process, methodologies, data sources, and documentation. Furthermore, providing personnel with the understanding of an AI system capabilities and the processes behind its actions, will also improve system reproducibility, allowing for seamless deployment across healthcare systems. *T*his is important for generative AI systems in healthcare because of their nature of being a black box system. This high-level understanding will help optimize trust, as operators will be aware of the capabilities and limitations of the AI systems they work with and know the appropriate settings for use^104^. With generative AI becoming more prevalent in healthcare, proper documentation is required to ensure all end users are properly educated on the capabilities and limitations of the systems they interact with. The generation process of generative AI models should be transparent. The references or facts should be provided together with answers and suggestions for clinicians and patients. Data sources used to train these models and the design procedures of these models should be transparent too. Furthermore, the implementation, deployment, and operation of these models need to be auditable, under the control of stakeholders in the healthcare setting.

### Privacy

Privacy is an assurance that the confidentiality of, and access to, certain information about an entity is protected^105^. Privacy is necessary in most military and medical applications of healthcare due to their confidential nature. Generative AI systems in healthcare must be HIPAA compliant for data disclosures, and secure to prevent breaches and developers should be advised how healthcare data should train systems for deployment. HIPAA compliance requires a regular risk assessment to determine how vulnerable patient data is ref. ^106^, thus a clinic utilizing generative AI systems in healthcare would have to determine if these systems are weak points in their technology network. For example, the utilization of generative AI models presents potential privacy breach risks, including prompt injection^107^, where malicious actions could be conducted by overriding an original prompt, and jailbreak^108^, where training data could be divulged by eliciting generated content. Furthermore, the capabilities of generative AI to process personal data and generate sensitive information make it crucial for these systems to be secure against data breaches and cyberattacks. Ensuring these systems are developed with data privacy and security in mind will assist in keeping protected patient information secure. Having these robust measures in place to maintain the privacy of the sensitive data collected and made by AI systems is crucial for the well-being of patients and for building trust with patients.

### Lawfulness

Lawfulness is the adherence to national and international law, including international humanitarian law and human rights law, as applicable^109^. This can be adopted for the use of generative AI in healthcare. The laws that must be adhered to are not laws of conflict, but rather those related to healthcare. Different states in the U.S. may establish different laws for AI systems that must be heeded for deployment in those areas^110^. Generative AI systems in healthcare also face legal challenges surrounding safety and effectiveness, liability, data privacy, cybersecurity, and intellectual property law^92^. A legal foundation must be established for the liability of action taken and recommended by these systems, as well as considerations for how they interact with cybersecurity and data privacy requirements of healthcare providers. Generative AI for healthcare must be developed with these legal challenges in mind to protect patients, clinicians, and AI developers from any unintended consequences.

### Empathy

Empathy is the ability to understand the personal experiences and emotions of another, without extensive bonding^111^. A principle for empathy is not directly referenced in any guidelines by the DOD or NATO. However, by emphasizing the need for human involvement in the treatment of patients, it is possible to create a framework for human involvement in generative AI applications to prevent gaps in accountability and ensure patients receive care that is empathetic and helpful^53^. There have been notable concerns about artificial empathy^112^ of chatbots, such as ChatGPT, reinforcing the need for a principle defining empathy for generative AI in healthcare^113^. An empathetic relationship between provider and patient brings several benefits to both the patient and the clinic treating them, such as better patient outcomes, fewer disputes with healthcare providers, higher patient satisfaction, and higher reimbursement^111^.

### Autonomy

Autonomy is the matter of control over one’s self and requires both freedom from controlling influences and the capacity for action for it to be maintained^114,115^. The more powerful AI systems become, the more concerns arise that humans do not control healthcare systems and care decisions^32^. Generative AI has seen staggering progress in the past several years, and hence, the protection of autonomy needs to be ensured when using generative AI in healthcare. Protecting human autonomy means that patients receive care according to their preferences and values and that clinicians can deliver treatment in the manner they want, without being encroached upon by the generative AI system. If autonomy in decision-making is not patient-focused, the potential for adverse events and poor clinical outcomes will surely follow^116^. By including provisions for protecting autonomy in using generative AI in healthcare, doctor-patient relations improve, and care quality is ultimately improved^117^.

## Conclusion

Generative AI has great potential to enhance and make high-quality healthcare more accessible to all, leading to a fundamental transformation in its delivery. Challenges posed by AI in healthcare often mirror those encountered in military. We propose the GREAT PLEA ethical principles, encompassing nine ethical principles, in the hope of addressing the ethical concerns of generative AI in healthcare, as well as the distinction between generative AI and “general” AI. This will be achieved by addressing the elevated risks mentioned previously in the paper. Generative AI necessitates guidelines that account for the risk of misinformation, ramifications of bias, and difficulty of using general evaluation metrics. Considering the widespread nature of generative AI and its risks, these ethical principles can protect patients and clinicians from unforeseen consequences. Following these principles, generative AI can be continuously evaluated for errors, bias, and other concerns that patients or caregivers may have about their relationship with AI in their field. The present moment urges us to embrace these principles, foster a closer collaboration between humans and technology, and effect a radical enhancement in the healthcare system.

These principles can be enforced through cooperation with lawmakers and the establishment of standards for developers and users, as well as a partnership with recognized governing bodies within the healthcare sector, such as the WHO or AMA.

We note that the enforcement of the proposed ethical principles, be it via evaluation approaches (e.g., Likert scale, prompting, or semantic similarity-based approaches for empathy^53,118,119^) or through other means, is out of the scope of this effort. As such, we acknowledge the lack of detailed enforcement procedures as the limitation of the work. At the same time, implementing AI metrics or enforcement methods for GREAT PLEA ethical principles can also be the potential future avenue for exploration.
